# Colorectal cancer patient-derived organoids and cell lines harboring *ATRX* and/or *DAXX* mutations lack Alternative Lengthening of Telomeres (ALT)

**DOI:** 10.1038/s41419-023-05640-3

**Published:** 2023-02-09

**Authors:** Marta Falcinelli, Giulia Dell’Omo, Elena Grassi, Elisa Mariella, Simonetta Maria Leto, Sharon Scardellato, Annalisa Lorenzato, Sabrina Arena, Andrea Bertotti, Livio Trusolino, Alberto Bardelli, Fabrizio d’Adda di Fagagna

**Affiliations:** 1 IFOM ETS, The AIRC Institute of Molecular Oncology, Milan, Italy; 2 Department of Oncology, University of Torino, 1060 Candiolo, Torino, Italy; 3 Candiolo Cancer Institute – FPO IRCCs, 10060 Candiolo, Torino, Italy; 4 Institute of Molecular Genetics IGM-CNR “Luigi Luca Cavalli-Sforza”, Pavia, Italy

**Keywords:** Telomeres, Telomeres

## Abstract

Telomere maintenance is necessary to maintain cancer cell unlimited viability. However, the mechanisms maintaining telomere length in colorectal cancer (CRC) have not been extensively investigated. Telomere maintenance mechanisms (TMM) include the re-expression of telomerase or alternative lengthening of telomeres (ALT). ALT is genetically associated with somatic alterations in alpha-thalassemia/mental retardation X-linked (*ATRX*) and death domain-associated protein (*DAXX*) genes. Cells displaying ALT present distinctive features including C-circles made of telomeric DNA, long and heterogenous telomeric tracts, and telomeric DNA co-localized with promyelocytic leukemia (PML) bodies forming so-called ALT-associated PML bodies (APBs). Here, we identified mutations in *ATRX* and/or *DAXX* genes in an extensive collection of CRC samples including 119 patient-derived organoids (PDOs) and 232 established CRC cell lines. C-circles measured in CRC PDOs and cell lines showed low levels overall. We also observed that CRC PDOs and cell lines did not display a significant accumulation of APBs or long telomeres with no appreciable differences between wild-type and mutated ATRX/DAXX samples. Overall, our extensive analyses indicate that CRC is not prone to engage ALT, even when carrying genetic lesions in *ATRX* and/or *DAXX*, and support the notion that ATRX/DAXX genomic footprints are not reliable predictors of ALT.

## Introduction

Telomere maintenance is a mechanism necessary to sustain cancer unlimited proliferation [1]. Most human cancers rely on re-expression of telomerase as telomere maintenance mechanisms (TMM). In a variable fraction of cancers, depending on tumor types, telomere maintenance is granted in a telomerase-independent manner by a mechanism known as alternative lengthening of telomeres (ALT). ALT involves a homologous recombination-dependent mechanism through which telomeric DNA is synthesized resulting in long and heterogeneous telomeres [2]. Although the expression of telomerase and ALT are the main TMM described in the literature, it has also been reported that tumors may display neither of the two TMM [3, 4]. In addition to long and heterogeneous telomeres, ALT cells have peculiar characteristics that include the accumulation of C-circles, which are extra-chromosomal partially single-stranded telomeric DNA circles, and ALT-associated promyelocytic leukemia (PML) bodies (APBs): nuclear structures where telomeric DNA, telomeric binding proteins and PML colocalize [2]. ALT tumors have been associated with somatic alterations in the alpha-thalassemia/mental retardation X-linked (*ATRX*) and the death domain associated protein (*DAXX*) genes, two chromatin remodeling factors [5]. In a systematic analysis of different cancer types, samples with *ATRX* alterations showed a reduction in *ATRX* expression and significantly longer telomeres [6]. In silico estimations from high-throughput sequencing data indicated that tumors with *ATRX* and/or *DAXX* alterations increased telomere content, a proxy for ALT identification [7, 8]. The correlation between *ATRX* and/or *DAXX* alterations and ALT would seem to predict ALT using short-read whole-genome sequencing and according to this, the genomic footprint of a tumor was proposed to predict the TMM [8]. However, recent meta-analyses revealed that predictions of ALT based on the *ATRX* and/or *DAXX* alterations do not correlate well with ALT phenotypes such as C-circles. This suggests that the analysis of *ATRX/DAXX* mutational status does not always guarantee a correct TMM classification [9].

ALT is frequent in tumors derived from mesenchymal tissues including osteosarcomas, liposarcomas, and tumors of central and peripheral nervous system or neuroendocrine system [10, 11]. On the contrary, ALT was reported to be relatively rare in other cancer types such as prostate, pancreas, and intestine tumors [10, 12]. Although in CRC there is evidence of telomerase re-expression, the prevalent TMM in this type of tumor has not been exhaustively probed with some contrasting reports on whether telomeres are shortened or maintained [13, 14].

Here, we investigated whether colorectal cancer (CRC) exhibits ALT features. We evaluated ALT markers in a large collection of CRC patient-derived organoids (PDOs) and cell lines. In order to accurately identify ALT cells, we implemented a deep phenotypic characterization including ALT markers such as C-circles accumulation, APBs, and telomere lengths distribution assessed by Fluorescent In Situ Hybridization (FISH). In particular, we characterized in depth CRC PDOs and cell lines harboring *ATRX* and/or *DAXX* mutations to probe the correlation between genetic alterations in these genes and ALT phenotypes.

## Materials and methods

### Cell culture

CRC cell lines were previously characterized by whole exon sequencing (WES) and RNA-seq [15, 16]. Each cell line was cultured in its specific media and grown at 37 °C in a 5% CO_2_ air incubator. Cell lines were routinely screened for mycoplasma contamination using the PCR Mycoplasma Detection Kit (ABM) according to the manufacturer’s protocol. The genetic identity of cell lines was ascertained by DNA typing using the PowerPlex 16 HS System (Promega), through Short Tandem Repeats (STR) at 16 different loci (D5S818, D13S317, D7S820, D16S539, D21S11, vWA, TH01, TPOX, CSF1PO, D18S51. D3S1358, D8S1179, FGA, Penta D, Penta E, and amelogenin). Amplicons from multiplex PCRs were separated by capillary electrophoresis (3730 DNA Analyzer; Applied Biosystems) and analyzed using GeneMapperID v.3.7 software (Life Technologies).

### Patient-derived organoids

CRC patient-derived organoids (PDOs) were generated from patients undergoing metastatic CRC surgical resection [17, 18]. Tumor specimens (0,5 cm × 0,5 cm) were shredded into small pieces and washed with PBS. Tumor fragments were suspended in a 3D extracellular matrix substitute (Matrigel®, Corning, or Basement Membrane Extract (BME), Biotechne) and dispensed onto 12-well plates as 200 µL droplets. After matrix polymerization (20 min at 37 °C), the droplets were overlaid with pre-warmed Dulbecco’s modified Eagle medium/F12 supplemented with penicillin-streptomycin, 2 mM l-glutamine, 1 mM n-Acetyl Cysteine, 1X B27, 1X N2 and 20 ng/ml EGF (Sigma-Aldrich). Organoids were tested for mycoplasma and maintained at 37 °C in a humidified atmosphere of 5% CO_2_.

### C-circle assay

Genomic DNA was extracted and purified by Maxwell DNA extraction kit. DNA concentration was measured by Qubit Assay and 40 ng DNA for the CRC cell lines and 150 ng DNA for PDOs were assayed for the presence of C-circles. C-circle assay (CCA) was performed as described in Henson et al., 2009 [19]. Briefly, samples were combined with BSA (10μl 10.2 mg/ml), 0.1% Tween, 1 mM each dATP, dGTP, dTTP, dCTP ϕ29 Buffer (1x) and 7.5U ϕ29 polymerase and incubated at 30° for 4 h, then 65° for 20 min. Reaction products were diluted in SSC 2x and dot blotted onto a Biodine B nylon membrane. DNA was UV-cross-linked onto the membrane and hybridized with an end-labeled ^32^P-(CCCTAA)_3_ probe at 37°. The membrane was exposed to a phosphorimager screen, and subsequently imaged on a Typhoon Imager (GE Healthcare, Chicago, IL, USA) and analyzed on ImageJ. The sample-specific background was obtained by running a parallel experiment omitting ϕ29 polymerase. Sample loading was normalized by probing against Alu DNA. A standard curve with U2OS DNA (from 80 ng to 5 ng) was used to calibrate signals. Data represent three or more independent experiments, the sample size (n) is specified for each graph in figure legends.

### ImmunoFISH

Cells were fixed with 4% PFA for 10 min and permeabilized for 10 min. Cells disaggregated from PDOs were permeabilized in 0.5% TritonX-100 for 30 min. Permeabilization was followed by 1 h blocking at room temperature. Nuclei were stained with 1 μg/ml 4’-6-Diamidino-2-phenylindole (DAPI, Sigma-Aldrich). Samples were incubated with anti-PML (PG-M3 SC-966 mouse monoclonal, Santa Cruz) and a hybridized with the Cy3-OO-(CCCTAA)_3_ telomeric PNA probe (Panagene) overnight at 4 °C. Cells were incubated with secondary antibody (anti-mouse 488, Alexa Fluor) for 45 min at room temperature and re-fixed in 4% PFA and 0.1% TritonX-100 for 10 min at room temperature to stably retain the bound primary and secondary antibodies and the standard DNA. Data represent three or more independent experiments, the sample size (n) is specified for each graph in figure legends.

### Imaging

Confocal inverted laser microscope (Leica TCS SP5) was used for imaging. The number and intensity of foci and dots per cell were analyzed by the imaging software ImageJ, using same settings for each sample in the same experiment. The number of APBs was counted as a number of co-localizations and normalized for the number of telomeres per field. Telomere intensity was measured as intensity per signal.

### Statistical analyses

Mean with Standard Error Mean (SEM) and individual values are shown in graphs. Statistical analysis was performed by one-way ANOVA or Kruskall–Wallis test, respectively for normally distributed and not normally distributed data. The variance was similar between the groups statistically compared (Levine’s test for homogeneity of variance). Dunett’s test was performed for multiple comparisons where each sample was compared with relative control as specified for each graph in figure legends. Statistical significance (*p* value) is mentioned for each graph in figure legends.

## Results and discussion

Previous reports indicated that telomeres can be shortened or maintained in CRC [20, 21]. Evidence of an ALT-dependent TMM in CRC has not been reported so far [12]. Here, we explored the engagement of ALT in CRC by assessing several individual markers in a collection of CRC PDOs and cancer cell lines. We first probed whether CRC harbors ALT-associated mutations in *ATRX* and/or *DAXX* genes by analyzing a collection of 119 CRC PDOs and 232 CRC cell lines previously characterized by whole exon sequencing (WES) or targeted sequencing. PDOs were characterized only for *ATRX* and we identified 7 ATRX mutated PDOs, while cell lines were analyzed for both *ATRX* and *DAXX* and we found 40 mutants for *ATRX*, 5 for *DAXX*, and 10 for both *ATRX* and *DAXX*.

**Fig. 1:**
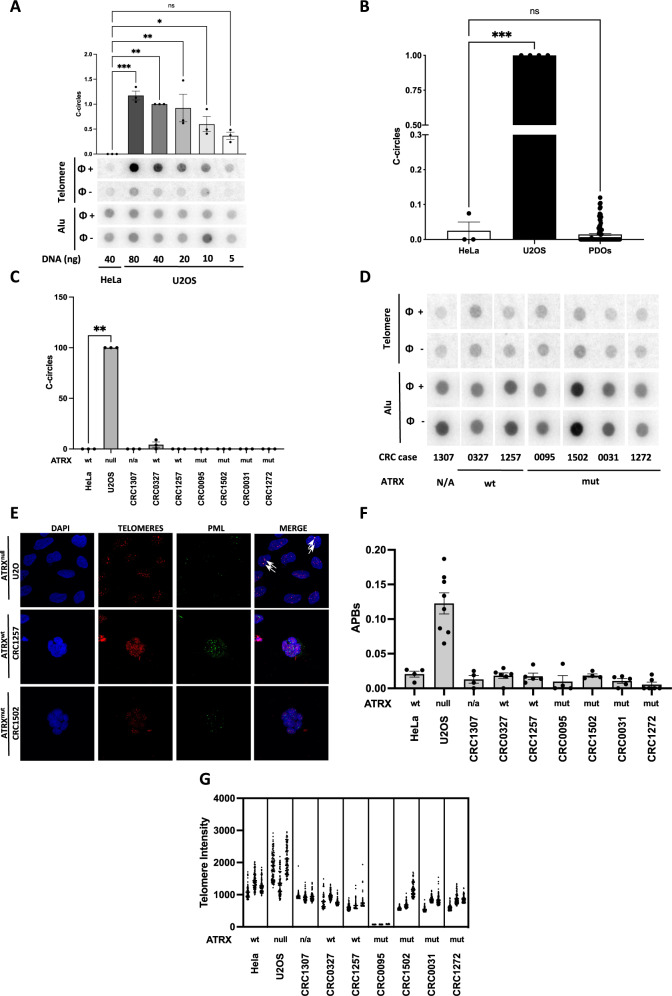
CRC PDOs do not display ALT markers. **A** C-circles assays (CCA, *n* = 3) were performed on 40 ng of genomic DNA extracted from ALT cells (HeLa) and on DNA (from 80 ng to 5 ng) extracted from ALT+ cells (U2OS). Representative dot blot is shown. Mean with SEM and individual values are shown. One-way ANOVA, Dunett’s multiple comparison test: each column compared with HeLa (***P < 0,001; **P < 0,002; *P < 0,033; ns=not significant). **B** 91 CRC PDOs were tested for C-circles (CCA, *n* = 91). Quantification was normalized on ALT+ cell line (U2OS, *n* = 4). Mean with SEM and individual values are shown. One-way ANOVA, Dunett’s multiple comparison test: each column compared with HeLa (*n* = 3), (***P < 0,001; ns = not significant). **C** CCA was carried out in triplicate (*n* = 3) on 7 (3 *ATRX*wt and 4 *ATRX*mut) selected PDOs. Quantification of the levels of C-circles is shown. Kruskall–Wallis test, Dunett’s multiple comparison test: each column compared with HeLa (**P < 0.002). **D** Representative dot blot for CCA on PDOs. Negative (HeLa) and positive (U2OS) controls are shown in (**A**). **E** Selected PDOs were studied for APBs and telomere intensity (*n* = 3). Representative images show cell nuclei (DAPI), telomeres and PML. **F** Number of APBs in cells from selected PDOs. Mean with SEM and individual values are shown. Kruskall–Wallis test, Dunett’s multiple comparison test: each column compared with HeLa**. G** Telomere intensity in selected PDOs as measured by FISH (*n* = 3). Each point represents a telomere. U2OS and HeLa cell lines were used as ALT+ and ALT- controls in all experiments, respectively.

C-circles assays (CCA) are robust ALT marker detection tests that can be implemented quantitatively in a relatively high-throughput fashion [22]. We validated CCA by performing a titration (from 80 ng to 5 ng) of DNA extracted from an ALT-positive (U2OS) cell line and comparing it to 40 ng of DNA extracted from an ALT-negative (HeLa) cell line. Quantification of the telomeric signals were normalized for input DNA on Alu signals and confirmed that, in our setup, C-circle levels were significantly higher in U2OS compared to HeLa cells (Fig. 1A), demonstrating the sensitivity and robustness of the assay. We thus performed CCA under the conditions described above on 91 CRC PDOs chosen from the original collection. We observed no significant C-circles accumulation in any of the PDO tested (Fig. 1B). To further explore ALT markers, we selected 7 PDOs from the original collection: 3 without known alterations in the ALT-associated gene *ATRX* and 4 with a non-synonymous mutation in *ATRX*. CCA performed in biological triplicate on such selected PDOs showed low levels of c-circles in both wild-type and mutated *ATRX* samples (Fig. 1C, D). We next evaluated APBs and telomere FISH signals intensity, an established method of individual telomeres length measurements, in cells obtained from PDO disaggregation. APBs were measured in U2OS and Hela as controls. In the PDO samples studied, in situ detections showed low numbers of APBs (representative images in Fig. 1E), comparable to those observed in ALT-negative control cells (HeLa) (Fig. 1F). Telomere length, assessed by FISH, showed generally short telomeric tracts in CRC cells derived from PDOs (Fig. 1G).

**Fig. 2:**
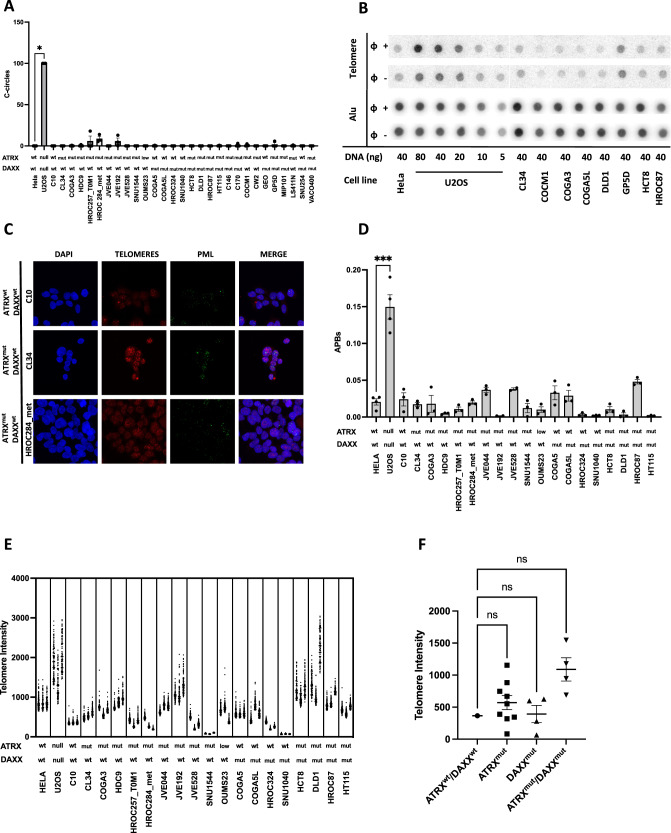
CRC cell lines do not display ALT markers. **A** CRC cell lines were tested for C-circles (CCA; *n* = 3). Quantification was normalized on ALT+ cell line (U2OS). Mean with SEM and individual values are shown. Kruskall–Wallis test, Dunett’s multiple comparison test: each column compared with HeLa (*P < 0.033). **B** Representative dot blot for CCA in cell lines. **C** Representative images of cell lines screened for telomere intensity and PML, nuclei stained by DAPI. **D** Number of APBs in selected cell lines (Hela *n* = 4; other cell lines *n* = 3). Mean with SEM and individual values are shown. Kruskall–Wallis test, Dunett’s multiple comparison test: each column compared with HeLa (***P < 0.001). **E** Telomere intensity evaluated in selected CRC cell lines by FISH (*n* = 3). **F** Telomere intensity in cell lines grouped for their ATRX and DAXX mutational status. Each point represents a cell line. Mean with SEM is shown for each group. One-way ANOVA, Dunett’s multiple comparison test: each column compared with ATRXwt/DAXXwt (ns= not significant). U2OS and HeLa cell lines were used as ALT+ and ALT- controls in all experiments, respectively.

We next extended ALT characterization to established CRC cell lines. We performed CCA in a subset of 30 CRC cell lines from the original collection. We observed low levels of C-circles in all samples with the exception of the ALT positive control (U2OS) (Fig. 2A, B). To test in greater depth whether *ATRX* and/or *DAXX* mutations had an impact on ALT features, we tested additional ALT markers in 19 cell lines selected from the original collection on the basis of their *ATRX* and *DAXX* mutational status. Specifically, this set of samples included an *ATRX* and *DAXX* wild type cell line (C10), 9 cell lines mutated for *ATRX*, 4 cell lines mutated for *DAXX*, and 4 cell lines mutated for both *ATRX* and *DAXX*. To test whether ALT phenotype might be influenced by *ATRX* expression levels, we also included a cell line (OUMS23) with low expression of *ATRX*. All samples were compared to the ALT-negative cell line (HeLa) and we used U2OS cell line as ALT positive control. All cell lines tested, with the exception of the positive control (U2OS), showed low number of APBs and their *ATRX*/*DAXX* mutational status was not associated with significant differences (Fig. 2C, D). Telomere length studied by FISH showed generally reduced signals patterns – the few exceptions were not accompanied by consistent changes in the other markers studied (APBs and c-circles) (Fig. 2E). Also, grouping telomere length results according to *ATRX* and *DAXX* mutational status did not highlight significant differences (Fig. 2F).

**Fig. 3:**
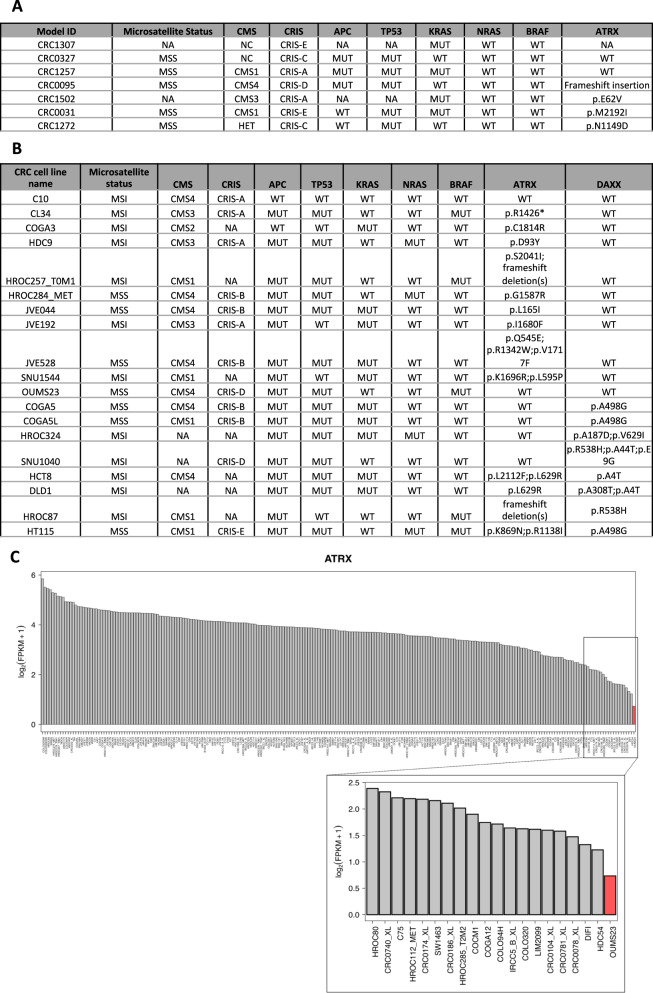
List of PDOs and cell lines selected for a further assessment of ALT markers. **A** The table lists the 7 PDOs studied for ALT markers, indicating microsatellite status, consensus molecular subtype (CMS), CRIS type together with mutational status in key oncogenes, tumor suppressors and the ALT-associated gene *ATRX*. **B** The table shows the 19 CRC cell lines characterized, indicating microsatellite status, consensus molecular subtype (CMS), CRIS type together with mutational status in key oncogenes, tumor suppressors and ATRX and DAXX alterations. **C** *ATRX* mRNA expression levels in CRC cell lines. OUMS23 cell line showed the lowest level of *ATRX* expression and was included in our screens.

Overall, our results show that both PDO from CRC patients and established CRC cell lines are not prone to engage ALT. This holds true also in PDOs and cell lines harboring mutations in *ATRX* and/or *DAXX* genes. These conclusions are based on a comprehensive analysis of ALT markers including C-circles, APBs, and telomere signals intensity. *ATRX/DAXX* mutations were proposed as ALT predictors [8]. This association was mainly based on genome sequencing-derived telomere content and correlated with telomere measurements by qPCR [7]. However, these predictions were not confirmed by further assessment (list of selected sample in Fig. 3A, B) of ALT phenotypical features [8, 9]. These limitations warned that ALT prediction based exclusively on genomic footprints of *ATRX* and/or *DAXX*, and also on *ATRX* expression levels (Fig. 3C) may overestimate or misclassify ALT tumors [9]. Our results, generated in an ample and well-characterized collection of PDO and cell lines, confirm that *ATRX*/*DAXX* mutational analyses are not reliable predictors of ALT. Our conclusions also support the importance of conducting multiple assays such as C-circles, APBs, and telomere intensity to accurately identify ALT tumors ensuring a correct TMM classification [23].

From a translational and clinical perspective, these conclusions suggest that the emergence anti-cancer therapies based on telomerase inhibition may be less of a concern in this tumor type [14, 24, 25]. Conversely, CRC may not be a suitable cancer type in which to explore the efficacy of proposed ALT-specific treatments [24, 26].

## Supplementary information

Reproducibility Checklist

## Data Availability

WES and targeted sequencing data from PDOs used in the current study are available in the European Genome-Phenome Archive (EGA) with the following study IDs: EGAS00001001305; EGAS00001001171. NGS data from cell lines used in the current study are available in the European Nucleotide Archive (ENA) with the following accession code PRJEB33045.
